# Diet and Nutrition for Hepatitis

**DOI:** 10.3390/nu13041210

**Published:** 2021-04-07

**Authors:** Takashi Himoto

**Affiliations:** Department of Medical Technology, Kagawa Prefectural University of Health Sciences, 281-1, Hara, Mure-Cho, Takamatsu, Kagawa 761-0123, Japan; himoto@chs.pref.kagawa.jp; Tel.: +81-87-870-1240

The impairment of liver function frequently causes various type of malnutrition, as the liver is one of the most important organs involved in maintaining nutritional homeostasis. Therefore, dietary or nutritional support can restore impaired liver function and improve the prognosis in patients with liver damage. This Special Issue contains nine peer-reviewed articles, including five original articles, which aim to establish novel nutritional interventions for various types of liver damage.

Hepatoprotective medicines are often prescribed to patients with drug-induced hepatitis. However, Nega et al. elucidate beneficial protective effects of probiotic *Bacillus* spores against acetaminophen-induced hepatic injury in mice [1]. *Bacillus* species have strong resistance to heat, toxic chemical compounds, and radiation. Thus, spore-forming probiotic bacteria are recognized as a promising alternative to *Bifidobacterium* and *Lactobacillus* strains. This study indicates that the probiotic *Bacillus* spores may make the intestinal barrier stronger and decrease the dissemination of microbial-derived compounds from the intestine, leading to an alleviation of endotoxemia.

The next two studies primarily focus on the efficacy of traditional medicines in experimental animal models of nonalcoholic fatty liver disease (NAFLD). NAFLD is one of the most common metabolic diseases, which is characterized by excessive lipid accumulation in the liver and the absence of alcohol consumption. Therefore, nutritional intervention may have a favorable effect in such patients. Liu et al. verify the utility of 14-deoxy-11,12-didehydroandrographolide (deAND), which is a major component of *Andrographis paniculata* (*A. paniculata*), in the experimental animal model of steatohepatitis [2]. *A. paniculata* is a kind of herb, and it is usually used as a traditional Chinese medicine for colds and diarrhea. It is of interest that the administration of deAND attenuates steatohepatitis in mice via the promotion of antioxidant and anti-inflammatory enzymes or proteins, suggesting that *A. paniculata* is a potential candidate for use in the treatment of NAFLD. On the other hand, the study by Nishiyama et al. largely highlights the alteration of gut microbiota in the experimental animal model of nonalcoholic steatohepatitis (NASH) after the administration of Bofutsushosan, a Japanese traditional medicine [3]. This medicine has often been prescribed to patients with obesity and obesity-related diseases. The authors demonstrate that the administration of Bofutsushosan formed a cluster of the gut microbiota that was significantly different from that in the control, and also resulted in an improvement in the biochemical liver enzymes and histological improvement in the liver, including reduced cellular lipoid accumulation, hepatocyte ballooning, and inflammatory cell infiltration. This medicine markedly increases the population of *Akkermansia muciniphila*, which has beneficial effects on various kinds of metabolic abnormalities, in the gut microbiota.

Savic et al. overview the clinical relevance of L-carnitine in patients with NAFLD. L-carnitine plays a crucial role in transporting fatty acids into mitochondria for β-oxidation and exporting excess acetyl-CoA from the mitochondrial matrix [4]. Therefore, L-carnitine deficiency is likely to impair fatty acid oxidation and subsequently cause triglyceride accumulation in the liver. Supplementation with L-carnitine may be a promising strategy as a novel treatment for NAFLD.

Recent advances in molecular biological technologies have enabled us to demonstrate that the alteration of genomics, epigenetics, microRNAs, and/or microbiota is involved in the development of nutritional disorders. Another goal of this Special Issue was to elucidate the novel mechanism of liver damage caused by the alteration of nutrition status, taking advantage of these molecular biological technologies. Mori et al. explore the effects of dietary iron overload on chemically induced liver injury in mice [5]. The dietary iron overload leads to iron deposits in the periportal hepatocytes of mice. Hepatic lipid peroxidation and hepatocellular DNA damage are increased in iron-overloaded mice that have been administrated with allyl alcohol, while they are decreased in iron-overloaded mice administrated with carbon tetrachloride (CCl_4_). These results imply that iron-overloaded livers may alter the susceptibility to drugs or chemical agents.

Kamada et al. document the clinical significance of the serum Mac-2 binding protein (M2BP) level in patients with fatty liver [6]. M2BP is a glycoprotein that contains seven potential N-glycosylations. Serum M2BP levels have been identified as a valuable hallmark for liver fibrosis. Surprisingly, serum M2BP levels were significantly correlated with serum triglyceride and alanine aminotransferase (ALT) levels, as well as body mass index (BMI), while they were negatively correlated with the high-density lipoprotein cholesterol (HDL-C) level in patients with fatty liver. However, the serum M2BP level was not significantly correlated with the FIB4-index, another indicator of liver fibrosis, in such patients. These data indicate that the synthesis of M2BP may be primarily associated with hepatic inflammation and an abnormality of lipid metabolism in the enrolled patients.

The review article by Zhou et al. overviews the impairment of autophagy and the initiation of endoplasmic reticulum (ER) stress in the pathogenesis of NAFLD [7]. The authors find that high fat and/or carbohydrate intake may trigger the impairment of autophagy and the initiation of ER stress in such patients. They also mention the putative mechanism by which high fat and/or carbohydrate consumption results in these phenomena.

The next review article by Uchida et al. reveals that oxidative stress has been recognized as a cancer-initiating response, while it can also become an anti-cancer agent that regulates the proliferation of cancer cells [8]. In the cancer stage, oxidative stress usually participates in a favorable response under treatment with molecular-target agents, such as sorafenib, regorafenib, or lenvatinib. This review article largely highlights these paradoxical effects of oxidative stress in the pathogenesis of chronic liver diseases, including carcinogenesis.

Finally, we summarize the current trends of essential trace elements in chronic liver diseases [9]. This review article primarily focused on the close correlation between the development of chronic liver diseases and metabolic abnormalities related to four essential trace elements: zinc, copper, selenium, and iron. We also introduce novel mechanisms by which an excess or deficiency of these trace elements leads to the development of chronic liver diseases.

In conclusion, this Special Issue provides many novel insights into how diet and nutrition can affect liver diseases for the readers of *Nutrients*. As a guest editor, I greatly appreciate the authors who submitted articles to this Special Issue and the reviewers who gave valuable feedback to the authors.
